# *Chlamydia trachomatis*-infected cells and uninfected-bystander cells exhibit diametrically opposed responses to interferon gamma

**DOI:** 10.1038/s41598-018-26765-y

**Published:** 2018-05-31

**Authors:** Joyce A. Ibana, Shardulendra P. Sherchand, Francis L. Fontanilla, Takeshi Nagamatsu, Danny J. Schust, Alison J. Quayle, Ashok Aiyar

**Affiliations:** 1 Immunopharmacology Research Laboratory, Institute of Biology, College of Science, University of the Philippines, Diliman, Quezon City, 1101 Philippines; 2 Department of Microbiology, Immunology and Parasitology, Louisiana State University Health Sciences Center, New Orleans, LA 70112 USA; 3 Department of Obstetrics and Gynecology, Faculty Medicine, University of Tokyo, Tokyo, Japan; 4 Department of Obstetrics, Gynecology, and Women’s Health, University of Missouri, Columbia, MO 15276 USA

## Abstract

The intracellular bacterial pathogen, *Chlamydia trachomatis*, is a tryptophan auxotroph. Therefore, induction of the host tryptophan catabolizing enzyme, indoleamine-2,3-dioxgenase-1 (IDO1), by interferon gamma (IFNγ) is one of the primary protective responses against chlamydial infection. However, despite the presence of a robust IFNγ response, active and replicating *C. trachomatis* can be detected in cervical secretions of women. We hypothesized that a primary *C. trachomatis* infection may evade the IFNγ response, and that the protective effect of this cytokine results from its activation of tryptophan catabolism in bystander cells. To test this hypothesis, we developed a novel method to separate a pool of cells exposed to *C. trachomatis* into pure populations of live infected and bystander cells and applied this technique to distinguish between the effects of IFNγ on infected and bystander cells. Our findings revealed that the protective induction of IDO1 is suppressed specifically within primary infected cells because *Chlamydia* attenuates the nuclear import of activated STAT1 following IFNγ exposure, without affecting STAT1 levels or phosphorylation. Critically, the IFNγ-mediated induction of IDO1 activity is unhindered in bystander cells. Therefore, the IDO1-mediated tryptophan catabolism is functional in these cells, transforming these bystander cells into inhospitable hosts for a secondary *C. trachomatis* infection.

## Introduction

*Chlamydia trachomatis* is the most common sexually transmitted bacterial pathogen, causing a myriad of diseases that can impinge severely on female fertility and the health of neonates. Under permissive physiological conditions, these obligate intracellular bacteria can invade, replicate, and establish long-term infections in the columnar epithelium of the urogenital tract, indicating their capacity to evade host immune responses. *C. trachomatis* has evolved many evasive strategies to promote its long-term survival within its human host. These include inhibition of apoptosis^1–3^, modulation of NFκB signaling^4,5^, downmodulation of CD1d^6^, and inhibition of transcription factors necessary for the expression of MHC classes I and II^7,8^.

Evidence from multiple animal studies indicates that a major host defense against *C. trachomatis* is mediated by IFNγ that is secreted by NK cells and T cells in the infected microenvironment^9–13^. IFNγ induces the expression of indoleamine-2,3-dioxygenase 1 (IDO1), an enzyme that catabolizes tryptophan to kynurenine^14,15^. By depleting tryptophan, IDO1 activation curtails the growth of *C. trachomatis*, which is a tryptophan auxotroph^16^. Under conditions of limited tryptophan availability, the chlamydial life-cycle deviates from normal development to form an aberrant, viable but non-cultivable, growth form termed persistence^17,18^. Renewal of tryptophan availability can reactivate persistent forms to return to normal development^19^. However, when initial levels of tryptophan are already limiting, IFNγ exposure can block all development, including the onset of persistence^20^. Curiously, although IFNγ inhibits the growth of *C. trachomatis* in cells that are pre-depleted of tryptophan *in vitro*, the developmental cycle of *C. trachomatis* serovar D exhibits a moderate resistance to the effects of IFNγ if the cytokine is added at the time of infection^21,22^. Consistent with the latter observations, clinical observations indicate the presence of active *C. trachomatis* infections within the infected endocervix despite the presence of higher than normal levels of IFNγ in the infected microenvironment^23^. Therefore, we wondered if *C. trachomatis* had a mechanism by which it could attenuate the effects of IFNγ secreted by immune cells in response to a primary infection.

Such a hypothesis is not without precedent. Many intracellular pathogens have evolved strategies to support their survival within their host cells by mitigating the host IFNγ response. A plethora of viral and bacterial effector molecules have been identified that interfere with the IFNγ-mediated activation of the JAK/STAT signaling pathway via a variety of mechanisms, including: 1) pathogen encoded proteins acting as decoys to block the IFNγ receptor (IFNGR) ligation^24^; 2) downmodulation of IFNGR expression^25^; 3) preventing activation of STAT1 by blocking its phosphorylation^26,27^; and 4) partially or fully inhibiting the nuclear translocation of activated STAT1^28–32^.

Despite possessing such mechanisms to evade the host IFNγ response, disseminated infections by many of these pathogens are prevented by the protective effects of IFNγ. It is likely these protective effects rely on the prevention of secondary infections. This is evidenced by multiple studies indicating that pre-treatment of host cells with interferons blocks the subsequent replication of a pathogen, even if it possesses effectors to evade such responses^33,34^. Therefore, while pathogens may circumvent the effects of interferons during primary infection, infection spread may be limited by the effects of interferons on uninfected bystander cells in the infected microenvironment. These observations may be relevant to chlamydial infections *in vivo*. Histological evidence from infected human tissues revealed a small number of infected cells to be surrounded by large numbers of uninfected-bystander cells^35,36^. Therefore, even if *C. trachomatis* possesses a mechanism to block the effect of IFNγ on an ongoing primary infection, the effects of the cytokine on bystander cells would block infection spread, consistent with overwhelming evidence that IFNγ is critical to control chlamydial infections *in vivo*.

We hypothesized that a differential effect of IFNγ on infected and bystander cells may explain the survival of *C. trachomatis* during a primary infection and its subsequent IFNγ-mediated clearance during a secondary infection. Therefore, to test our hypothesis, we investigated the effects of *C. trachomatis* on the IFNγ-mediated induction of IDO1 along the JAK/STAT cell signaling pathways using a robust method that can distinguish between the effects of IFNγ on *C. trachomatis*-infected and bystander cells. Our approach revealed an exciting mechanism by which *C. trachomatis* attenuates the IFNγ-mediated IDO1 induction, and clarified the role of bystander cells in the host IFNγ response during *C. trachomatis* infection

## Results

### CPP-labeling of *C. trachomatis* elementary bodies permits the recovery of pure populations of live infected and bystander cells by flow cytometry

**Figure 1:**
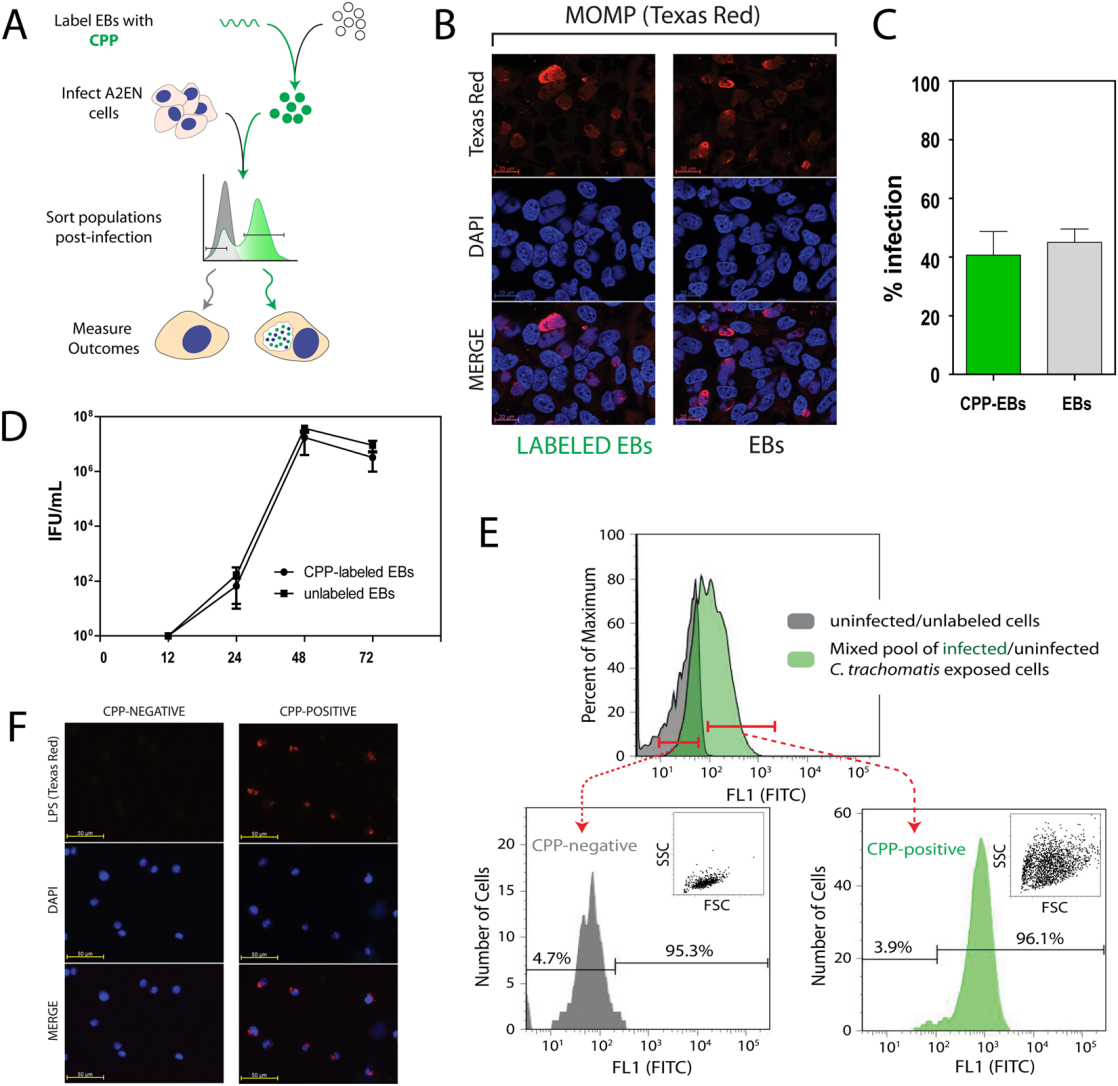
Isolation of pure populations of live infected and bystander cells using fluorescently-labeled EBs. (**A**) A schematic representation of the labeling and isolation protocol. A2EN cells were exposed to CPP-labeled *C. trachomatis* EBs, and subsequently separated into infected and bystander pools by flow cytometry. (**B**) MOMP staining (Texas Red) performed at 38 hpi reveal labeled EBs to form inclusions in A2EN cells that are indistinguishable from control EBs. Cells were counterstained with DAPI, and images were acquired using a 40 × objective. (**C**) Equal IFUs for labeled and control EBs infected equivalent fractions of exposed A2EN cells indicating that labeling does not affect infectivity. Infection was quantified by LPS staining. (**D**) Growth curves of control and labeled EBs after infections of A2EN cells indicate that infections initiated using labeled EBs develop at the same rate as those initiated with control EBs. IFU recovery at the indicated times post-infection were measured by infection of HeLa 229 cells. (**E**) The upper panel shows flow cytometric analyses of mock-infected A2EN cells (gray histogram), and A2EN cells exposed to *C. trachomatis* (green histogram). *Chlamydia*-infected cells were sorted into CPP-positive and CPP-negative pools using the gating strategy indicated by the red markers. The lower panels display histograms indicating the sorting efficiency. 95% of CPP-positive and CPP-negative pools display fluorescence profiles that lie within the gates used to isolate them. (**F**) The purity of sorted cells was examined by LPS-staining the sorted populations. >95% of the CPP-positive cells were infected as indicated by LPS-positivity. Similarly, >95% of CPP-negative cells were also LPS-negative. The insets in the lower panels indicate differences in the FSC/SSC profiles of CPP-positive and CPP-negative cells. The former are large and more granular consistent with their containing inclusions.

The Cell Penetrant Peptide’s (CPP’s) sequence and the EB labeling procedure used are described in the Methods section. Briefly, as schematically depicted in Fig. 1A, after a short co-incubation with the CPP, labeled EBs were separated from unincorporated peptide by centrifugation and used to infect the endocervical cell-line A2EN. Punctate FITC signal, presumed to be EBs, were observed around the periphery of A2EN cells by one-hour post infection (hpi). By 36 hpi, inclusions with internal and peripheral FITC signal were observed. Pertinently, by this time, no fluorescent signal was observed at the cell periphery of infected or uninfected cells (Supporting Information Figure S1). This result was interpreted to indicate that the CPP had penetrated EBs or was tightly associated with them prior to infection. It also indicated that CPP-labeling did not disrupt infection or inclusion formation.

Before using the CPP as a tool to isolate pools of infected and bystander cells, we tested whether it affected *C. trachomatis* development or infectivity. Intracellular staining at 38 hpi using an antibody against the chlamydial major outer membrane protein (MOMP) revealed no difference in inclusion size (Fig. 1B) or number (Fig. 1C) when labeled EBs were compared to unlabeled EBs. Similar results were obtained when cells were stained for chlamydial lipopolysaccharide (LPS) (data not shown). A growth curve after infection using labeled EBs was performed to quantify IFU recovery between 12-72 hpi (Fig. 1D). The results indicated that infections initiated using labeled EBs developed at the same rate as control infections (*ibid*). Therefore, CPP-labeling does not affect the infectivity or normal development cycle of *C. trachomatis* in A2EN cells.

CPP-labeled EBs were used to separate a mixed population of cells exposed to *C. trachomatis* into pools of infected and uninfected bystander cells using the protocol outlined in Fig. 1A. A2EN cells exposed to *C. trachomatis* were harvested and passed through a fluorescence activated cell sorter to obtain separate pools of labeled and unlabeled cells (Fig. 1E).

The histogram overlay in the upper panel of Fig. 1E shows that infection with labeled EBs results in a mixed population of cells with a wide range of fluorescent intensities (green profile), many of which can be clearly distinguished from unlabeled control cells (gray profile). The gating strategy shown in this panel was used to obtain FITC-positive and FITC-negative populations that are indicated in the lower panels of Fig. 1E. These sorted populations were sorted a second time to evaluate their purity, and were found to be approximately 95% pure, i.e. 95% of each sorted population lay within the gates used to obtain that population. Forward and side scatter profiles of these populations indicated that the FITC-positive cells were larger and more granular, consistent with their containing inclusions (insets in the lower panels of Fig. 1E).

To evaluate the *Chlamydia*-infection status of FITC-negative and FITC-positive cells, sorted cells were fixed and subjected to intracellular LPS staining. Staining results indicated that >95% of the FITC-positive cells were LPS-positive; likewise, >95% of the FITC-negative cells were also LPS-negative (Fig. 1F). Therefore CPP-labeled EBs can be used to sort a mixed pool of *C. trachomatis*-exposed cells into pools of live infected and bystander cells that are >95% pure.

### Analyses of sorted populations indicate that the IFNγ-induced expression of IDO1 is attenuated in infected cells

**Figure 2:**
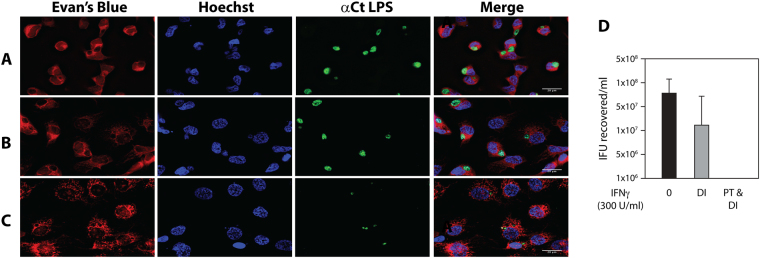
IFNγ-treatment beginning with infection does not inhibit normal development of *C. trachomatis* and IFU recovery at 48 hpi. (**A**) A2EN cells were infected with *C. trachomatis*, fixed, and stained for LPS at 24 hpi. Coverslips were counter-stained with Evan’s Blue and Hoechst dye. (**B**) A2EN cells were infected and processed as in “A”, with the exception that IFNγ (600 U/mL) was added during the time of infection (DI). (**C**) A2EN cells were pre-treated (PT) with IFNγ (300 U/mL). Infection, post-infection conditions, and processing were as in “B”. Images were obtained using a 63X objective. The scale bar indicates a distance of 20 μm. (**D**) IFUs recovered at 48 hpi after infection of A2EN cells using the IFNγ-exposure conditions in panel A “0”, panel B “DI” (During Infection), and panel C “PT (Pre-treatment) & DI”. IFU recovery was measured by infection of HeLa 229 cells. The difference in IFU recovery between control conditions “0”, and when IFNγ was added at the start of infection “DI” was not significant (p > 0.2). No recoverable IFUs were detected in pre-treated cells “PT & DI”.

The IFNγ-induced enzyme, indoleamine-2,3-dioxygenase-1 (IDO1), catabolizes tryptophan into kynurenine, and is proposed to be the primary mediator of the IFNγ-induced protective response against chlamydial infection. Pre-treatment of epithelial cells with IFNγ at low concentrations results in tryptophan depletion and the induction of persistent chlamydial growth forms^17^. While characterizing the effect of IFNγ on chlamydial growth in A2EN cells at 38 hpi, it was observed to not substantially impair infection or normal development if it was added at the time of infection (Fig. 2A,B). Consistent with these observations, IFU recovery 48 hpi after infection of A2EN cells indicated a modest decrease when IFNγ was added at the time of infection (Fig. 2D). In contrast, no recoverable IFUs were obtained at 48 hpi when A2EN cells were pre-treated with IFNγ (Fig. 2C,D). These observations are consistent with published reports that *C. trachomatis* serovar D infections of HeLa cells are moderately resistant to the effects of IFNγ^21^. Therefore, we considered it possible that *C. trachomatis* interfered with the induction of IDO1 by IFNγ, or with IDO1 function. This hypothesis was explored by using the CPP to isolate pure populations of live infected and bystander cells after culture in the presence or absence of IFNγ.

**Figure 3:**
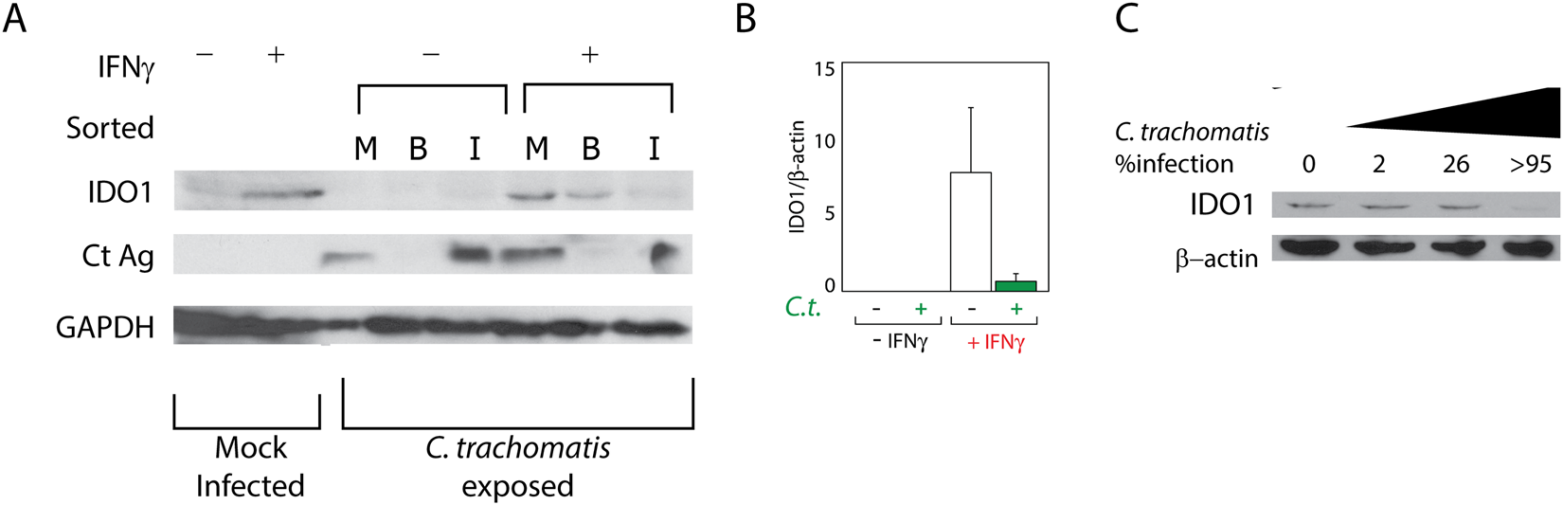
IFNγ-induced expression of IDO1 is attenuated in infected but not bystander cells. (**A**) A2EN cells were exposed to *C. trachomatis* after which IFNγ (600 U/mL) was either not added “−”, or added “+”. Exposed cells were sorted into bystander “B” or infected “I” cells at 38 hpi. “M” refers to the initial mixed unsorted pool of *C. trachomatis*-exposed cells. IFNγ induced the expression of IDO1 in mock-infected cells. Induced expression was also observed in the mixed and bystander pools exposed to IFNγ. IFNγ-induced IDO1 expression was greatly reduced in purified *C. trachomatis*-infected cells. Sorting purity was evaluated using a polyclonal antibody against chlamydial EBs, which detects a protein that migrates ~17 kD. This protein was detected only in the mixed pool and purified infected cell pool. Immunoblots against GAPDH were used as a loading control. (**B**) mRNA isolated from purified bystander and infected cells cultured in the presence or absence of IFNγ (600 U/mL) was used in qPCRs to quantify IDO1 expression. IDO1 mRNA levels were below the detection limit in the absence of IFNγ. In the presence of IFNγ, bystander cells expressed ~12-fold higher levels of IDO1 mRNA relative to the levels observed in infected cells (set to 1). qPCRs for β-actin mRNA were used for normalization. Statistical significance calculated using Student’s t-test is indicated. (**C**) Exposure of A2EN cells that resulted in the infection of 2%, 26% and >95% of A2EN cells at 24 hpi in the presence or absence of IFNγ (600 U/mL).

After infection with CPP-labeled EBs, A2EN cells were cultured in the absence or presence of IFNγ. At 38 hpi, these cultures were separated into pools of infected (CPP-positive) and bystander (CPP-negative) cells that were tested for the expression of IDO1 by immunoblot (Fig. 3A). As anticipated, IFNγ exposure induced IDO1 expression in mock-infected cells and in the mixed pool of unsorted cells. Interestingly, the level of IDO1 protein was lower in IFNγ-exposed infected cells relative to bystander cells (*ibid*). The infection state of the two sorted populations was confirmed using a polyclonal anti-chlamydial EB antibody. Purified infected cells were positive for a ~17 kD chlamydial antigen that was also detected in the unsorted mixed pool of cells, but not in mock-infected or purified bystander cells (*ibid*).

Quantitative PCR analysis using mRNA isolated from purified infected and bystander cells indicated that the lower levels of IDO1 protein within IFNγ-exposed infected cells correlated with a lower IDO1:β-actin mRNA ratio in these cells (Fig. 3B). When normalized to β-actin mRNA levels, IFNγ-exposed bystander cells had ~8-fold higher levels of IDO1 mRNA than infected cells (*ibid*). The level of IDO1 mRNA was below the detection limit in the absence of IFNγ exposure.

The differential expression of IDO1 in bystander and infected cells after IFNγ exposure was recapitulated using infections at increasing IFU/mL, which resulted in the infections of 2%, 26% and >95% in A2EN cells. After IFNγ exposure, a strikingly diminished level of IDO1 protein was detected by immunoblot when > 95% of A2EN cells were infected, but not when smaller fractions of cells were infected (3C). No difference in β-actin expression was observed under all three conditions indicating no broad down-regulation of host cell protein levels can be attributed to chlamydial infection. Finally, annexin V/propidium iodide staining indicated that neither the high infection rate nor IFNγ exposure induced apoptosis of A2EN cells at 24 hpi, the time at which the analyses in Fig. 3C were conducted. Therefore, the decreased levels of IDO1 protein detected when >95% of A2EN cells are infected cannot be attributed to cellular apoptosis or necrosis (data not shown).

When considered together, the results in Fig. 3 support a model in which IFNγ inefficiently induces IDO1 within *Chlamydia*-infected cells, relative to the induction of IDO1 in bystander cells.

### STAT1 is retained in the cytoplasm of IFNγ-exposed infected cells

The mechanism by which IFNγ induces IDO1 expression is well defined. Briefly, association of IFNγ with its receptor (IFNGR) results in the activation of STAT1 by the JAK1/JAK2 kinases. Activated STAT1 translocates to the nucleus, where it transactivates expression of the IDO1 gene whose promoter contains cognate STAT1 binding sites^37–40^. The sharply reduced levels of IDO1 mRNA within infected cells led us to test whether *C. trachomatis* infection affected this signaling cascade.

**Figure 4:**
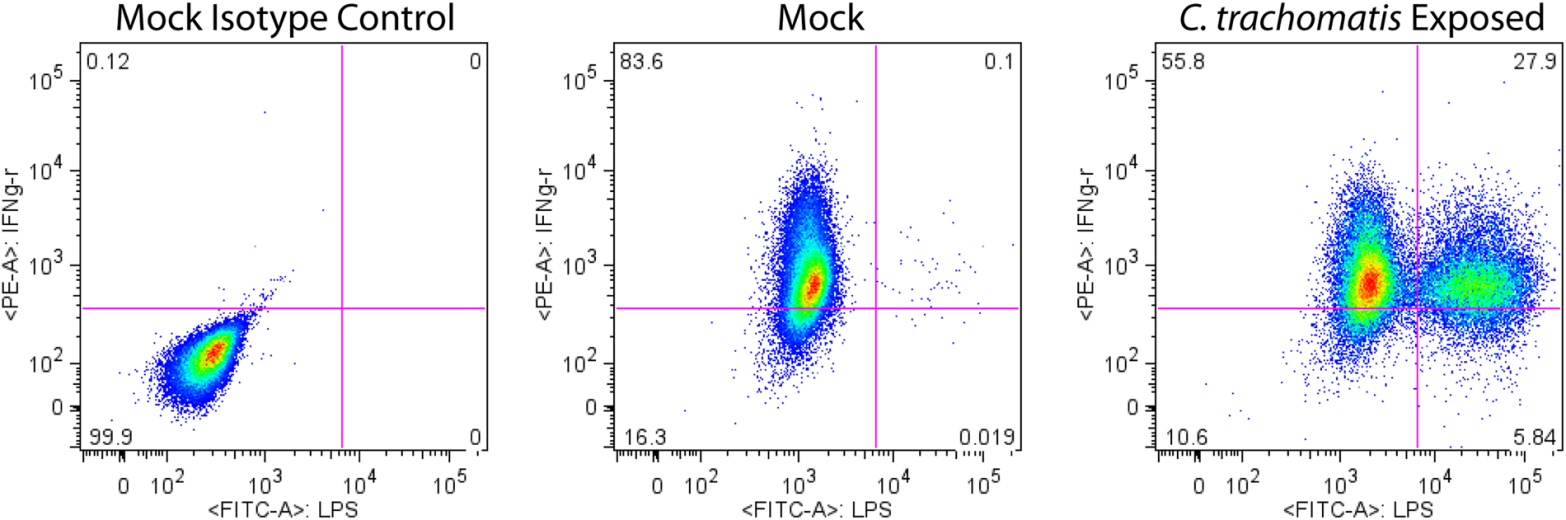
*C. trachomatis* infection does not decrease the surface expression of IFNGR in A2EN cells. Mock-infected or *C. trachomatis*-exposed A2EN cells were harvested and stained for surface IFNGR. After this, they were fixed, permeabilized, and stained for LPS. Bystander cells are IFNGR + /LPS−, while infected cells are double-positive. Cells were stained at 24 hpi. LPS staining intensity is shown on the X-axis, and IFNGR staining intensity is on the Y-axis.

The intracellular bacterial pathogen, *Listeria monocytogenes*, attenuates IFNγ signaling by down-modulating IFNGR^25^. Therefore, we used flow cytometry to quantify the level of IFNGR on the surface of infected and bystander A2EN cells. Multiple experiments indicated no reduction in the surface expression of IFNGR on *C. trachomatis* infected cells relative to bystander or mock-infected cells, in the presence of IFNγ (Fig. 4).

**Figure 5:**
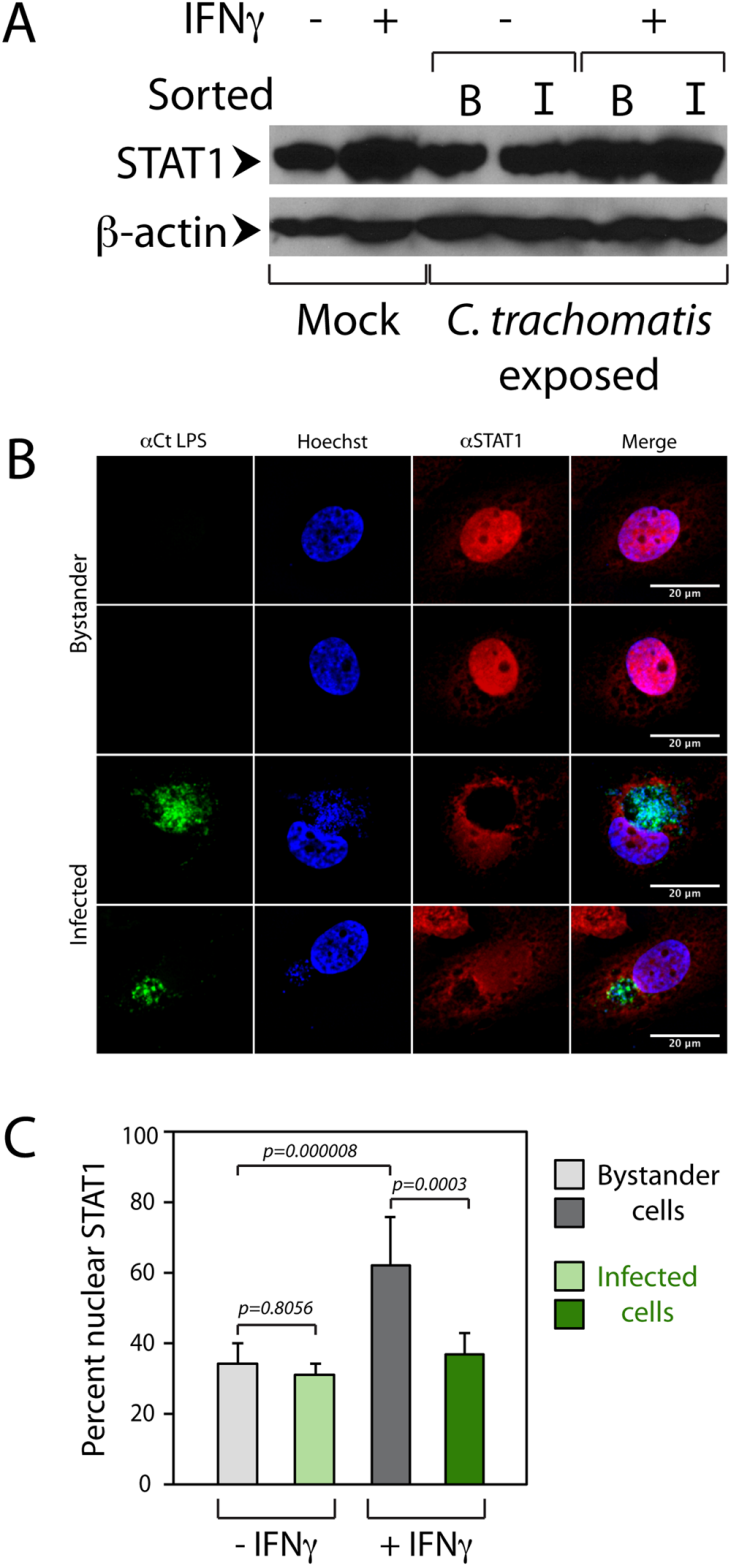
*C. trachomatis* inhibits IFNγ-induced STAT1 nuclear translocation. (**A**) STAT1 protein levels were examined by immunoblot in mock-infected, bystander “B”, and infected “I” cells in the absence “−” or presence “+” of IFNγ (600 U/mL). IFNγ treatment induced STAT1 levels in bystander and infected cells. A β-actin loading control is shown. (**B**) IFNγ efficiently induces STAT1 nuclear translocation in bystander but not infected cells. A2EN cells were plated on coverslips and exposed to IFNγ (600 U/mL) beginning at the time of infection. Coverslips were fixed and stained for STAT1 and LPS at 38 hpi. Hoechst dye was used for counter-staining. Examples of two bystander and infected cells are shown. The entire fields containing these cells are shown in Supporting Figure S4, along with experiments conducted in the absence of IFNγ. Images were obtained using a 63X objective. The scale bar indicates a distance of 20 μm. (**C**) The percent nuclear STAT1 in infected and bystander cells was calculated as described in the Methods section. Exposure to IFNγ dramatically increases the level of nuclear STAT1 in bystander cells, but not infected cells. Statistical significance as calculated using the Wilcoxon rank-sum test is indicated.

STAT1 degradation is another mechanism used by some intracellular pathogens to down-modulate host cell IFNγ-dependent responses^41^. However, immunoblot analyses examining STAT1 levels showed no difference between infected and bystander cells in the presence or absence of IFNγ (Fig. 5A). Therefore, *Chlamydia* does not attenuate IDO1 expression by down-modulating IFNGR or depleting STAT1 through cleavage or degradation.

The intracellular distribution of STAT1 affects its capacity to be activated and consequently function as a transactivator. STAT1 must be accessible to the JAK1/JAK2 kinases for it to be phosphorylated in response to IFNγ. After its phosphorylation, activated STAT1 must translocate to the nucleus to activate IFNγ-responsive promoters. For this reason, aberrations in the sub-cellular distribution of STAT1 prior to and after IFNγ exposure are predicted to affect IDO1 expression. Therefore, we examined the sub-cellular distribution of STAT1 in bystander and infected cells in the absence or presence of IFNγ. As reported for other cell types^42^, STAT1 displayed a broad nucleo-cytoplasmic distribution when A2EN cells were cultured in the absence of IFNγ (Supporting Figure S2). Addition of IFNγ caused STAT1 to accumulate in the nuclei of bystander but not infected cells (Fig. 5B, Supporting Figure S2). We quantified the effect of IFNγ on STAT1’s distribution in nuclear and cytoplasmic compartments using z-projections of deconvolved stacks as described in the Methods section. After IFNγ exposure approximately 60% of the total cellular STAT1 localized to the nuclei of bystander cells (Fig. 5C). In contrast, the bulk of total STAT1 remained cytoplasmic within IFNγ-exposed infected cells (*ibid*).

### STAT1 nuclear translocation, but not its phosphorylation, is blocked in IFNγ-exposed infected cells

The impaired nuclear translocation of STAT1 within IFNγ-exposed infected cells is consistent with the reduced expression of IDO1 observed in these cells. IFNγ-induced STAT1 nuclear import requires its phosphorylation^43,44^. Phosphorylation of a specific tyrosine residue, corresponding to Y701 in STAT1α, induces an allosteric change necessary for its nuclear import^45,46^. The failure to observe nuclear accumulation of STAT1 within IFNγ-exposed infected cells led us to query its phosphorylation status in these cells.

**Figure 6:**
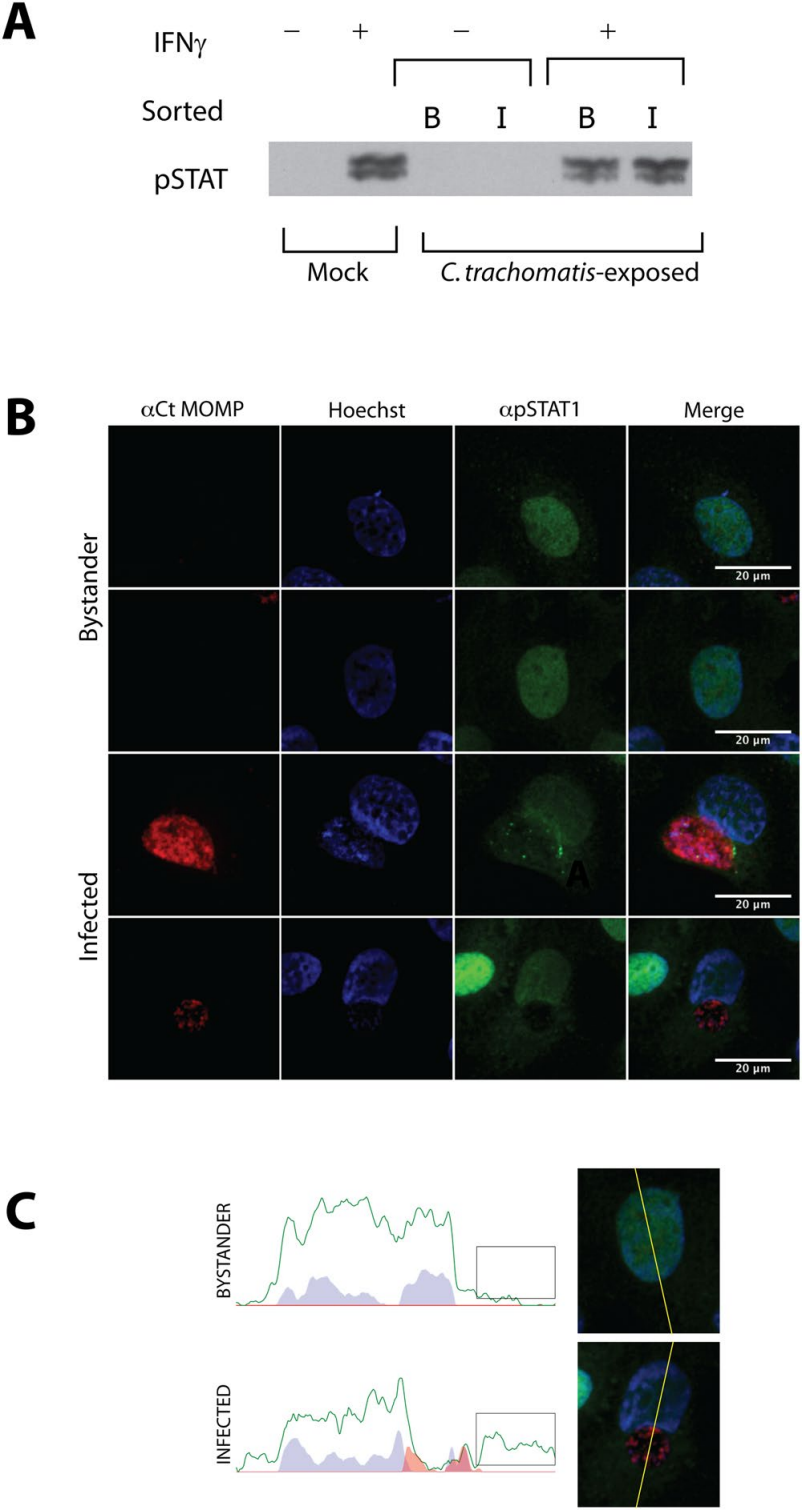
IFNγ induced pSTAT1 nuclear translocation is attenuated in infected cells. (**A**) The immunoblot shown in Fig. 5A was stripped and probed with a rabbit monoclonal antibody specific for Y701 phosphorylated STAT1 (pSTAT1). pSTAT1 was observed in mock-infected, bystander and infected cells exposed to IFNγ (600 U/mL), indicated as “+”, but not in the absence of IFNγ-exposure. (**B**) *C. trachomatis*-exposed cells cultured in the presence of IFNγ (600 U/mL) for 38 hpi were stained for pSTAT1 with the rabbit anti-pSTAT1 antibody used for the immunoblots shown in panel A. Cells were also stained with an antibody against MOMP and counterstained with Hoechst dye. Representative cells indicating pSTAT1 localization in bystander and infected cells are shown. Images were obtained using a 63x objective. The scale bar indicates a distance of 20 μm. The fields containing these cells along with control fields are shown in Supporting Figure S3. (**C**) The distribution profiles for pixel densities corresponding to pSTAT1 (green profile), Hoechst dye (filled blue profile) and MOMP (filled red profile) along a marker line are shown for a bystander cell and an infected cell. The boxed region indicates cytoplasmic pSTAT1 pixel density.

Immunoblots performed using a rabbit monoclonal antibody specific to Y701-phosphorylated STAT1 (pSTAT1) yielded a surprising result. It was anticipated that diminished nuclear accumulation of STAT1 in IFNγ-exposed infected cells would correspond to lower pSTAT1 levels; in contrast, there was no difference in the levels of pSTAT1 in bystander and infected cells after exposure to IFNγ (Fig. 6A). The specificity of this antibody is indicated by the absence of pSTAT1 in cells not exposed to IFNγ (*ibid*). The total STAT1 levels increased after exposure to IFNγ, corroborating previous findings^47^, and pSTAT1 levels increased in bystander and infected cells after IFNγ exposure to similar extents. Therefore, it appeared likely that nuclear import of pSTAT1 was reduced or inhibited in *Chlamydia*-infected cells.

Indirect immunofluorescence microscopy using the rabbit monoclonal antibody against pSTAT1 was used to assess its nucleo-cytoplasmic distribution. Examples of bystander and infected cells exposed to IFNγ are shown in Fig. 6B; and the entire fields containing these cells are shown in Supporting Figure 3. As observed earlier, pSTAT1 was primarily nuclear in bystander cells, but not infected cells. Paralleling observations with STAT1, 63% of the total cellular pSTAT1 was nuclear in bystander cells, whereas 35% was nuclear in infected cells. This analysis, conducted using infected and bystander cells within 10 microscopic fields, indicated the difference to be statistically significant (p < 0.005). The differential distribution of pSTAT1 can be observed when the pixel density profile for pSTAT1 is plotted along a marker line drawn across the cell. The majority of pSTAT1 signal (green line) in bystander cells lies within the bounds of the nuclear signal (filled blue profile). In contrast, both nuclear and cytoplasmic pSTAT1 signals can be observed within infected cells; for example, the pSTAT1 signal to the right of the inclusion signal (filled red profile) is highlighted by a box (Fig. 6C).

We decided to corroborate this observation using a different antibody by immunofluorescence microcopy after exposing cells to IFNγ. Direct immunofluorescence experiments conducted using the Pacific Blue-conjugated antibody revealed pSTAT1 to be present in the nuclear/perinuclear regions of bystander cells. In contrast, pSTAT1 was broadly distributed within infected cells (Supporting Figure S4). Confirming immunoblot analyses, no pSTAT1 was detected in either bystander or infected cells in the absence of IFNγ (*ibid*).

### Uninfected bystander cells are primarily responsible for the IFNγ-mediated protective host response to *C. trachomatis* infection

The observation that IFNγ-driven responses are attenuated within infected cells led us to posit that IFNγ-mediated protective responses in the host resulted from the effect of IFNγ on bystander cells. Successful spread of a *Chlamydia* infection requires normal bacterial replication within the initially infected cells, release of EBs, and their subsequent infection of surrounding cells. Our observation that IFNγ-dependent responses, such as the induction of IDO1, are not affected in bystander cells suggests a critical role for these cells in limiting the spread of infection. Our model is also supported by previous observations that pre-exposing cells to IFNγ inhibits *C. trachomatis* development more effectively than when IFNγ exposure is begun concurrent with infection^21^.

**Figure 7:**
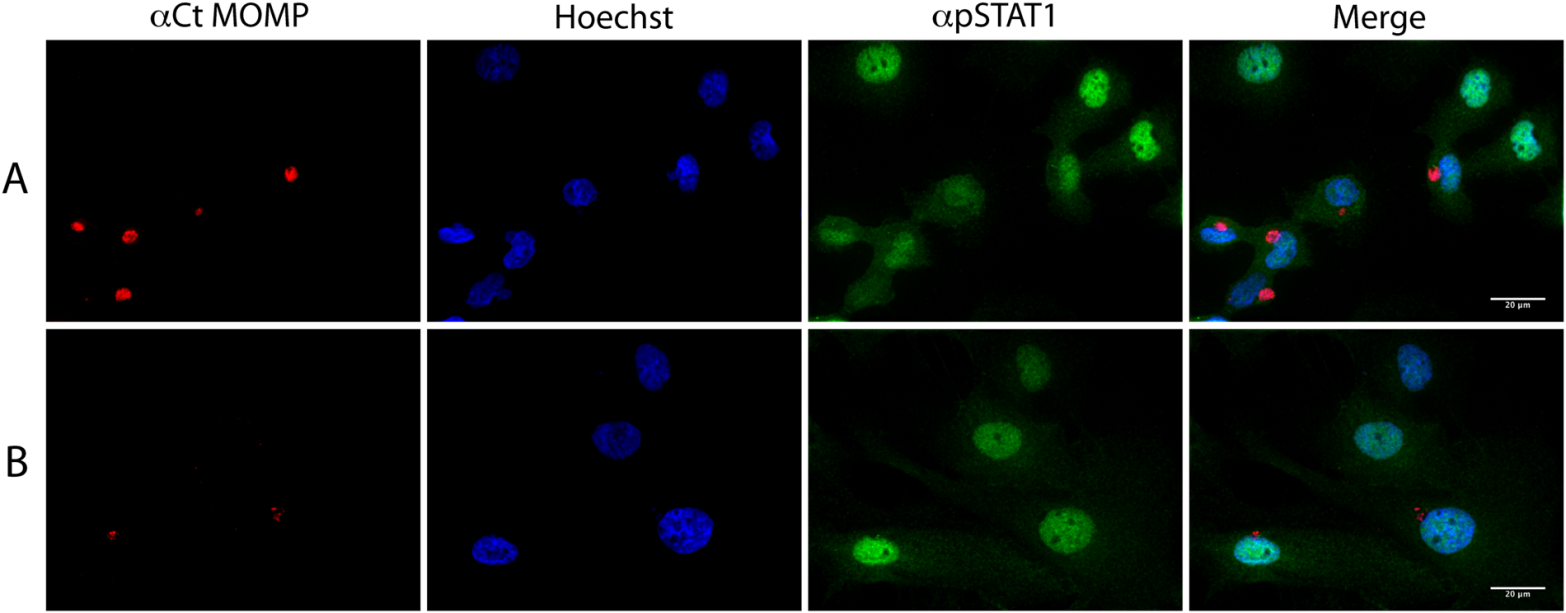
*C. trachomatis* infection does not disrupt the nuclear localization of pSTAT1 in cells pre-treated with IFNγ. (**A**) *C. trachomatis*-exposed cells were treated with IFNγ (600 U/mL) starting at the time of infection were fixed at 24 hpi and stained for pSTAT1 and MOMP. Cells were counter-stained with Hoechst dye. As observed earlier, pSTAT1 nuclear localization is attenuated within infected cells of which an example is shown. No attenuation is observed in bystander cells. (**B**) Cells were pre-treated with IFNγ (300 U/mL) for 24 hours, then infected and maintained in IFNγ (600 U/mL) for 24 hpi. Processing and staining was performed as described for panel A. Under these conditions, pSTAT1 was observed to be nuclear within infected cells, of which an example is shown here, and also bystander cells (shown in Supporting Figure S9). Images were obtained using a 63X objective. The scale bar indicates a distance of 20 μm.

Differences in tryptophan availability are critical to the magnitude of IFN gamma–related inhibition of chlamydial growth^17,48^ Chlamydial infections conducted in the presence of cell culture media containing lower basal levels of tryptophan allow for enhanced inhibition of chlamydial growth by IFN gamma^49,50^ when compared to infections conducted in culture media containing higher tryptophan levels^21^ In fact, in order to detect the inhibitory effects of IFN gamma on chlamydial replication when using media containing substantial amounts of tryptophan, earlier investigators were required to pre-deplete the media of tryptophan^51^. Results presented in Fig. 7A show that *C. trachomatis* can develop in A2EN when IFNγ is added at the time of infection but not when cells are pretreated of IFNγ prior to infection. This is consistent with the finding shown in Fig. 2B which indicate an absence of recoverable IFU when A2EN cells are exposed to IFNγ prior to and during infection, rather than only during infection.

Because IFNγ blocks spread of a primary infection, a corollary to our model predicts that chlamydial infection will not disrupt the nuclear localization of pSTAT1 activated by IFNγ prior to infection. We tested this by comparing the nucleo-cytoplasmic distribution of pSTAT1 at 24 hpi, when IFNγ was added to cells concurrent with infection, or 24 hours prior to infection and then maintained during infection. The outcome of this experiment, shown in Fig. 7B, supports this prediction. As observed previously, *Chlamydia* infection attenuated nuclear accumulation of pSTAT1 when IFNγ was added at the time of infection. In contrast, pSTAT1 was observed to be nuclear if cells were pre-exposed to IFNγ for 24 hours prior to infection and maintained in IFNγ during infection. These experiments reveal that the effect of IFNγ on bystander cells is critical to limit spread of a primary infection.

## Discussion

**Figure 8:**
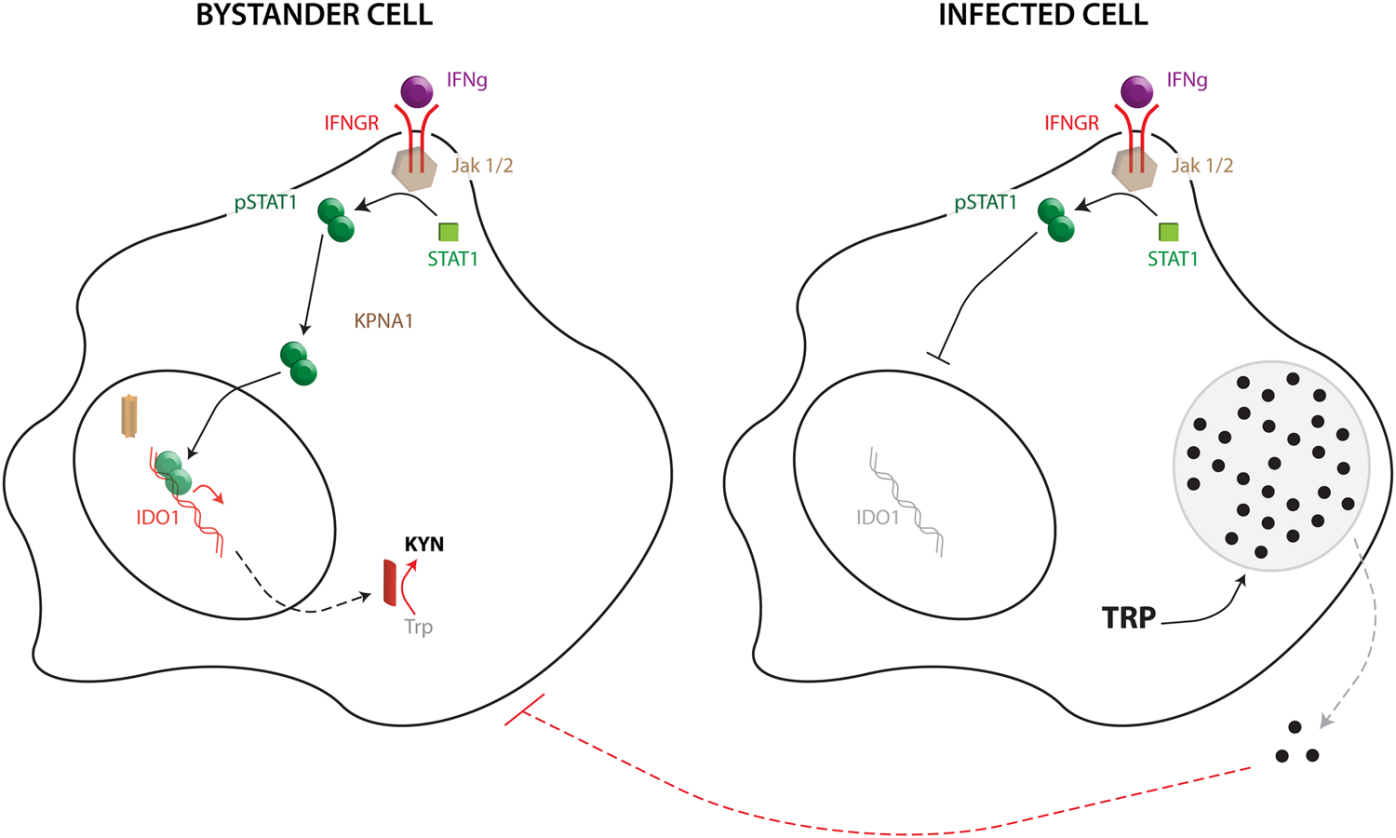
A model describing the effects of IFNγ on bystander and infected cells. The IFNγ-induced expression of IDO1 is not affected in bystander cells. However, inability to translocate activated STAT1 into the nucleus, results in attenuated induction of IDO1 within infected cells. Therefore, although IFNγ has a limited effect on primary infection, the activation of IFNγ-induced protective responses in bystander cells restricts the spread of infection.

We have described a novel method to fluorescently label *C. trachomatis* EBs without reducing their infectivity or normal development after infection. This method has permitted us to recover pure populations of infected and bystander cells from a mixed pool of host cells exposed to *Chlamydia*. Results obtained with these pools have uncovered a critical difference between infected and bystander cells in their response to the protective cytokine, IFNγ. While unanticipated, it was observed that the IFNγ-induced expression of IDO1 is attenuated specifically in infected cells, but not bystander cells. Observations made using sorted populations prompted us to examine the mechanism by which IDO1 induction is impaired within infected cells. Our findings lead us to propose the model diagrammed in Fig. 8.

Exposure of cells to *C. trachomatis* does not disrupt the IFNγ-dependent induction of IDO1 via the JAK-STAT pathway in uninfected bystander cells. In contrast, this pathway is functionally disrupted within infected cells without discernable effects on: 1) the surface expression of IFNGR; 2) STAT1 levels, or 3) STAT1 phosphorylation. Instead, *Chlamydia* interferes with IDO1 induction by disrupting pSTAT ability to translocate into the nucleus.

Our studies have also revealed a critical role for bystander cells in controlling infection. IDO1 induction in response to IFNγ is not affected in bystander cells permitting tryptophan depletion in the infection microenvironment. Therefore, the effects of IFNγ on bystander cells creates an environment that is not conducive to the development of secondary infections, thereby limiting spread of a primary infection. A role for bystander cells in controlling chlamydial infections is underscored by histological evidence from infected human tissues indicating a small number of infected cells to be surrounded by large numbers of uninfected bystander cells^36^.

The critical difference we have uncovered in the response of infected and bystander cells to IFNγ was obscured when studies examining protein expression and mRNA levels were conducted using an unsorted population of cells exposed to *Chlamydia*. Such differences between bystander and infected cells are not unique to the IFNγ response. Previous studies examining cell surface proteins indicated that the NK cell ligand, MIC-A, is upregulated on infected but not bystander cells^52^. Therefore, while we have used it to probe responses to IFNγ, our approach will be of general utility to discern differences between infected and bystander cells. Currently, studies requiring a pool of mostly-infected cells rely on infections performed with an m.o.i that is sufficiently high to infect the majority of cells exposed to *Chlamydia*. Indeed, our finding that the IFNγ-induced expression of IDO1 is suppressed within infected cells was confirmed using conditions in which >95% of *Chlamydia*-exposed cells were infected. This suppression was not observed when only 25% of cells in the exposed pool were infected. Although infections using high m.o.i have been informative, they do not reveal the contributions of bystander cells toward host defenses against infection. This is especially relevant because histological examination of *Chlamydia*-infected animal and human tissues reveal a small number of infected cells to be surrounded by large numbers of uninfected bystander cells^35,36,53^.

Observations on several intracellular bacterial pathogens have revealed that far from being inert observers, bystander cells play a critical role in controlling infection. A single cell analysis to evaluate the NFκB-dependent induction of IL-8 after infection by *Shigella flexneri* uncovered dramatic differences between infected and bystander cells^54^. Although the NFκB-dependent induction of IL-8 was downmodulated in *Shigella* infected cells, NFκB activation and IL-8 expression were amplified in uninfected bystander cells (*ibid*). Protective immune responses dependent on bystander cells have also been described for the intracellular pathogens *Listeria monocytogenes* and *Salmonella typhimurium (ibid)*. While they differ mechanistically, these findings coincide with our observations that the effect of IFNγ on bystander cells limits the spread of a primary *Chlamydia* infection *in vitro*. Bystander cells are also likely to mediate proinflammatory responses associated with *C. trachomatis* pathology. Paradoxically, although there is strong evidence that NFκB responses are inhibited in *Chlamydia*-infected cells *in vitro*^4,5,55^, animal and human studies indicate that a proinflammatory response is a hallmark of *C. trachomatis* pathogenesis [reviewed in^56^]. Akin to *S. flexneri*, bystander cells in the *C. trachomatis*-infected microenvironment may drive this proinflammatory response. From a broader perspective, studies with multiple intracellular pathogens emphasize the need to elucidate bystander cell contributions to infection dynamics and outcomes. The method we have developed to isolate live infected and bystander cells provides a powerful tool to investigate the mechanisms by which bystander cells modulate the *Chlamydia* infection microenvironment.

The mechanism by which *Chlamydia* creates a distinction in the effect of IFNγ on infected and bystander cells may be relevant for other nuclear proteins, such as NFκB, that are functionally disrupted during infection [4,5]. The observation that NFκB-dependent transcriptional responses are downmodulated within infected cells was previously attributed to its cleavage by the chlamydial CPAF^5^ and/or TSP proteases^4^. Recent studies in which infected cell extracts were prepared under conditions where CPAF was inactive during cell lysis indicated that the p65 subunit of NFκB is not cleaved by a chlamydial protease within intact cells^57^. Therefore, although NFκB activity is reduced within infected cells, this reduction can no longer be attributed to its cleavage by a chlamydial protease. A reduction in NFκB activity would also be observed if the capacity of *Chlamydia* to block nuclear accumulation of activated STAT1 extended to NFκB. A possible block to NFκB nuclear import in *Chlamydia*-infected cells is not without precedent. Enteropathogenic *E. coli* and *Shigella* secrete T3 effectors that prevent the nuclear translocation of NFκB^58^.

Like *Chlamydia*, multiple other intracellular pathogens have developed mechanisms to mitigate the effects of IFNγ on infected cells. The inhibitory mechanisms used by these pathogens fall into four broad categories. The first involves downmodulation of IFNGR, which renders *Listeria monocytogenes-*infected macrophages refractory to IFNγ^25^; The second uses STAT1 degradation as exhibited by *Leishmania donovani*^41^. The third category, inhibition of STAT1 activation, is used by *Chikungunya* virus^26^. The fourth involves the inhibition of activated STAT1 nuclear import and can be seen in infections by Ebola virus^28^, porcine reproductive and respiratory syndrome virus^30^ and severe acute respiratory syndrome coronavirus^32^. The Ebola virus expressed protein, VP24, binds the same domain of KPNA1 as STAT1. Association of VP24 with KPNA1 displaces STAT1, thereby preventing its nuclear localization^28^. The Nsp1b protein of porcine reproductive and respiratory syndrome virus disrupts STAT1 nuclear localization by degrading KPNA1^30^. The ORF6 protein of the SARS coronavirus inhibits STAT1 nuclear localization by a mechanism that has not been completely elucidated. ORF6 sequesters karyopherin α2 and it is proposed that sequestration affects the cellular environment necessary for KPNA1 function^32^.

While the identity of the chlamydial effector that blocks pSTAT is not described in this study we would like to highlight our findings that the nuclear import of activated STAT1 is not blocked in uninfected bystander cells adjacent to infected cells. Our powerful strategy to examine pure populations of infected and uninfected bystander cells permitted the resolution of this phenomenon, which is otherwise masked in mixed-populations of cells that were exposed to *C. trachomatis* infection. While identifying this effector is not within the scope of this study, it enticing to hypothesize that, when identified, it may represent an excellent therapeutic target whose inhibition is predicted to increase the susceptibility of a primary *Chlamydia* infection to protective host cytokine responses.

## Experimental Procedures

### Sequence and synthesis of the fluorescein-conjugated cell penetrant peptide

The cell penetrant peptide (CPP) used corresponds to the first AT-hook domain (a.a. 31–55) of the Epstein-Barr nuclear antigen 1 protein of Epstein-Barr virus preceded by one additional arginine residue. With the exception of glycine, all other amino-acids in the CPP are in the D-configuration. Fluorescein was covalently linked to the C-terminus. The amino-terminus of the CPP is amidated. The conjugate was synthesized by Peptide 2.0, and purified to >95% purity via HPLC. Purity was confirmed by HPLC using methods described previously^59^.

### Cell culture and infections

The endocervical epithelial cell line (A2EN) described previously was utilized in this study^60^. A2EN cells were initially propagated in antibiotic-free keratinocyte serum-free medium (KSFM) supplemented with 30 μg/mL recombinant epidermal growth factor, 0.1 ng/mL bovine pituitary extract and 0.4 mM CaCl_2_ (Sigma). A derivative of A2EN was generated by conditioning the cells to grow in Dulbecco’s minimal essential medium with 5% fetal bovine serum. A2EN clones were generated by seeding cells at a probability of 0.33 cells/well in a 96-well plate. Clones were evaluated for a homogenous response to IFNγ as measured by nuclear localization of STAT1. A2EN clone A17 was used for the experiments described here.

*C. trachomatis* serovar D (D/UW-3/Cx) was used for all experiments. *C. trachomatis* was propagated on HeLa 229 cells. Highly-purified elementary bodies (EBs) were obtained from infected HeLa cell lysates after two rounds of differential density centrifugation using Iodixanol (Optiprep^TM^, Sigma Aldrich) step-gradients as described previously^61^. The multiplicity of infection (m.o.i) used on A2EN cells corresponded to IFUs determined using HeLa 229 cells as described previously. A2EN cells were infected with *C. trachomatis* in SPG (10 mM sodium phosphate [pH 7.2], 0.25 M sucrose, 5 mM L-glutamic acid) at a multiplicity of infection (m.o.i) of 3 based on IFU calculations conducted on HeLa cells that resulted in infection rates of about 40% in A2EN cells, unless otherwise stated. Mock infections using SPG alone were included in each experiment.

### Fluorescent labeling of *C. trachomatis* elementary bodies

The fluorescently tagged CPP was added at a final concentration of 1 μg/mL to 10^8^ IFUs of purified EBs in PBS at a final volume of 500 μL. After incubation at 37 °C for 10 minutes, EBs were separated from unincorporated CPP by centrifugation at 13,000 × g for 5 minutes at 4 °C. The CPP-labeled EB pellet was re-suspended in SPG at an appropriate dilution for infection. The infectivity of labeled EBs was assessed by comparing the percentage of *C. trachomatis* LPS-positive cells after infections using equal amounts of CPP-labeled EB and unlabeled EB preparations from the same stock. Infectivity was also assessed by quantifying the IFU recovered at 12, 24, 48, and 72 hours post-infection of HeLa 229 cells with CPP-labeled and unlabeled EBs.

### Fluorescence microscopy of CPP-labeled *C. trachomatis*

A monolayer of A2EN cells grown on Type 1 coverslip inserts in 12-well culture plates were infected with CPP-FITC labeled EBs. Cells were fixed with 4% paraformaldehyde, at 1, 38, and 42 hours post infection (hpi), permeabilized with permeabilization/fixation reagent (BD Biosciences), and counterstained with DAPI (Molecular Probes). Coverslips were mounted on microscope slides using Prolong Gold antifade reagent (Invitrogen).

To confirm infection and evaluate the purity of sorted cells, 50 μL aliquots of CPP-negative and CPP-positive sorted cells were spotted on microscope slides, air-dried, and fixed with ice-cold methanol. Cells were permeabilized, stained with chlamydial-LPS antibody (Accurate) that was detected using a secondary antibody conjugated to Texas Red (Jackson Labs). Nuclei were counterstained with DAPI. The fraction of LPS-positive cells to DAPI-positive cells was used to calculate the percent of infected cells as well as the purity of the sorted bystander and infected cells using ImageJ.

### Flow Activated Cell Sorting (FACS)

To obtain pure populations of *C. trachomatis*-infected and uninfected-bystander cells, A2EN cells were infected using CPP-labeled EBs. Mock and *C. trachomatis*-infected A2EN cells were harvested using Accutase (Chemicon) and re-suspended in PBS. The cell suspension was passed through a 70 micron nozzle at a flow rate range of 20–100 events/second in a BD FACS Aria for sorting. The fluorescence intensities of uninfected and unlabeled cells were used to set a threshold of positivity for CPP-labeled cells. To confirm the purity of the sorted cell populations, sorted cells from the negative and positive channels were evaluated by flow cytometry a second time to determine the percentage of CPP-positive and CPP-negative cells. Cell size and granularity of each population was assessed by plotting forward and side scatter profiles. The purity of sorted populations was also evaluated by LPS staining as described above. For protein analyses, sorted cells were collected in PBS. For RNA analyses, sorted cells were collected in tubes containing RNAlater® (Applied Biosystems), and stored in −80 °C until use.

### Protein analyses by immunoblotting

Mock and *C. trachomatis*-infected A2EN cells were cultured in media alone or media containing 600 U/ml IFN gamma for 24 or 38 hours post-infection. Cells were sorted into infected and bystander populations at these times, and processed for immunoblotting as described previously^62^. Briefly, sorted cells were counted using a Beckman Z1 Coulter counted and pellets. Cell extracts were made at an equivalence of 5 × 10^4^ cells/μl. Sample load equivalency was confirmed by re-probing stripped membranes using antibodies against the housekeeping genes GAPDH or β-actin. Protein bands were detected using chemiluminescence. Membranes were re-probed using other relevant antibodies after being stripped using Restore Plus (Thermo-Fisher). Probing with a rabbit anti-chlamydial EB antibody detects a clear protein band that runs along a 17kD MW marker. This has been shown previously as a useful marker for the presence of *Chlamydia* in different cell lysate preparations^63^ Primary and secondary antibodies used for protein detection are described in Table 1.

**Table 1:** Antibodies used for Immunoblotting.

Protein/antigen	Primary antibody	1° dilution	Secondary antibody	2° dilutions
IDO1	Mouse monoclonal clone 10.1 (Millipore)	1:500	Goat anti-mouse IgG (H + L)-HRP (Jakson ImmunoResearch)	1:3000
STAT1	Rabbit polyclonal C-24 (SC-345) (Sta. Cruz Biotechnology)	1:500	Donkey anti-rabbit IgG-HRP (GE Healthcare)	1:5000
pSTAT1 (pTyr701)	Rabbit monoclonal (S.213.5) (Thermo Scientific)	1:1000	Donkey anti-rabbit IgG-HRP (GE Healthcare)	1:3000
GAPDH	Mouse monoclonal (sc-47724) (Sta. Cruz Biotechnology)	1:1000	Goat anti-mouse IgG (H + L)-HRP (Jakson ImmunoResearch)	1:3000
β-actin	Mouse monoclonal clone AC-15 (Sigma)	1:10,000	Goat anti-mouse IgG (H + L)-HRP (Jakson ImmunoResearch)	1:3000
*C. trachomatis* EBs	Rabbit Polyclonal antibody (Thermo Scientific)	1:500	Donkey anti-rabbit IgG-HRP (GE Healthcare)	1:5000

### Quantitative real-time PCR for IDO1 mRNA

*C. trachomatis*-exposed cultures were sorted into CPP-negative and CPP-positive cell populations in collection tubes containing RNAlater. The samples were then processed for quantitative PCR for IDO1 expression, normalized to β-actin, as previously described^62^. Statistical analyses were performed using Student’s t-test.

### Flow Cytometry for IFNGR expression

The expression of IFN gamma receptor (IFNGR) were assessed in mock-infected cells and *C. trachomatis*-exposed A2EN cells by flow cytometric analyses. The population of infected and bystander cells were delineated by immunostaining the cells with FITC-conjugated anti-Chlamydial-LPS antibody. IFNGR expression were quantified using a PE conjugated anti-IFNGR. Details of antibodies used are described in Table 2. The immuno-stained A2EN cells were assessed using FACS Calibur and data were analyzed using Flowjo analysis software (TreeStar).

**Table 2:** Antibodies used for flow cytometry and immunofluorescence microscopy.

Protein/antigen	Primary antibody	1° dilution	Secondary antibody	2° dilutions
Chlamydial-LPS	Mouse monoclonal-FITC (clone 1683) (YVS1683) (Accurate)	1:35	None (1° conjugated to FITC)	None
Chlamydial-MOMP	Mouse monoclonal (HT10) (SC-51837) (Sta. Cruz Biotechnology)	1:50	Alexa Fluor 568 goat anti-mouse IgG2a (Invitrogen)	1:300
*C. trachomatis* EBs	Rabbit Polyclonal antibody (Thermo Scientific)	1:1000	Alexa Fluor 568 anti-rabbit IgG (Invitrogen)	1:200
STAT1	Mouse monoclonal-PE (clone 1/Stat1) (BD Biosciences)	1:100 (FC)	None (1° conjugated to PE)	None
	Rabbit polyclonal (clone C-24) (SC-345) (Sta. Cruz Biotechnology)	1:100	Alexa Fluor 647 donkey anti-rabbit IgG (H + L) (ThermoFisher Scientific)	1:200
pSTAT1 (pTyr701)	Mouse monoclonal pacific blue (clone 14/P-STAT1) (BD biosciences)	1:50 (IF)	None (1° conjugated to pacific blue)	None
	Rabbit monoclonal (S.213.5) (Thermo Scientific)	1:200	Alexa Fluor 647 donkey anti-rabbit IgG (H + L) (ThermoFisher Scientific) or Alexa Fluor 488 goat anti-rabbit IgG (H + L) (Invitrogen)	1:200
IFNR-PE	Human monoclonal (clone GIR-94) (BD Pharmingen)	1:20	None (1° conjugated to PE)	None

### Indirect immunofluorescence microscopy

A2EN cells were seeded on Type 1 glass coverslips in 12-well dishes. Upon reaching a confluence of ~70–80%, the cells were mock or *C. trachomatis*-infected at an m.o.i of 3. Cells were fixed with 4% paraformaldehyde at 24 or 40 hpi, and processed for immunostaining using the primary and secondary antibodies for proteins being assessed. Antibodies used are described in Table 2. Pre-chilled acetone-methanol (1:1) was used to permeabilize cells for STAT1 or phosphorylated STAT1 detection. To detect phospho-STAT1, cells were fixed using pre-warmed fixation buffer (BD Biosciences) for 10 minutes at 37 ^°^C. The cells were then permeabilized using chilled Perm Buffer III (BD Biosciences) for 30 minutes at -20 ^°^C. Nuclei and inclusions were counterstained using DRAQ5 (Thermo Scientific), or Hoechst 33342 as described previously. Wide-field fluorescence images were acquired using an inverted Zeiss AxioObserver AX10 microscope, equipped with an AxioCam MRm camera, and controlled using AxioVision version 4.6.3. Images were acquired using the 20X, 40X and 63X objectives. Only on images acquired with the 63X objective (N.A. = 1.4) were quantified. For immunofluorescence, acquisition times were determined using a positive control, and kept constant for all samples in the same experiment. Minimally, five fields were acquired per slide, and slides from at least two independently performed experiments were used to quantify experimental outcomes.

### Image processing, deconvolution, and quantification

In a preliminary experiment, image stacks containing 100 z-sections spaced 0.1 microns apart were acquired using cells stained with Evans Blue and Hoechst dye. These images were deconvolved using AxioVision’s Nearest Neighbor algorithm. Thresholding was used to delineate the circumference of each nucleus. Using 10 cells, the average cell depth was estimated to be 7.4 microns in the area corresponding to the nuclear circumference. Non-thresholded stacks of the 10 nuclei indicated the average nucleus height to be 6.1 microns. After determining these values, all images were acquired as stacks containing 35 z-sections spaced 0.2 microns apart. Acquired images were processed using Fiji (ImageJ)^64^. Image stacks for each channel were deconvolved using emission wavelength-specific theoretical point spread functions (PSFs) that were generated using the ImageJ plugin, PSF Generator^65^. The Landweber deconvolution algorithm implemented in the Image J plugin DeconvolutionLab was used for deconvolution^66^. Thirty-five positively-constrained iterations of deconvolution were performed with no early stopping criteria.

Deconvolved stacks corresponding to the central 60% of the nucleus (18 slices) were used to evaluate nucleo-cytoplasmic signal distribution. For this, a region of interest (ROI) corresponding to each nucleus within the field was created by thresholding. When applied to the deconvolved stack, these ROIs were used to obtain pixel density information for each nucleus across the stack using ImageJ’s Multi Measure function. Pixel density distribution across the stack was used to select 18 z-sections to create an average intensity z-projection for that nucleus. Average intensity z-projections of the same 18 z-sections were made using deconvolved stacks for the other channels. The z-projected images were used to quantify pixel densities in ROIs corresponding to the area of the entire cell or just the nucleus. To determine nucleo-cytoplasmic distribution, the pixel density of the nucleus ROI was divided by the pixel density of the cell area ROI.

Pixel density profiles shown in Fig. 6C were made by drawing a marker line across the cell. This was applied as an ROI to each channel, after which ImageJ’s Plot Profile function was used to provide pixel density values along the line. These were imported into Microsoft Excel and graphed.

## Electronic supplementary material

Supplementary Figures
