# Supplementary material for: Chlamydia trachomatis-infected cells and uninfected-bystander cells exhibit diametrically opposed responses to interferon gamma
Source: Sci Rep. 2018 May 31;8:8476. doi: 10.1038/s41598-018-26765-y (PMC5981614; doi:10.1038/s41598-018-26765-y)
Supplement: Supplementary file 1 — Supplementary Figures [file 41598_2018_26765_MOESM1_ESM.pdf]

Chlamydia trachomatis-infected cells and uninfected-bystander cells exhibit diametrically opposed responses to interferon gamma

Joyce A. Ibana[1]*, Shardulendra P. Sherchand[2], Francis F. Fontanilla[1], Takeshi Nagamatsu[3], Danny J. Schust[4], Alison J. Quayle[2] and Ashok Aiyar[2]

SUPPLEMENTARY FIGURES

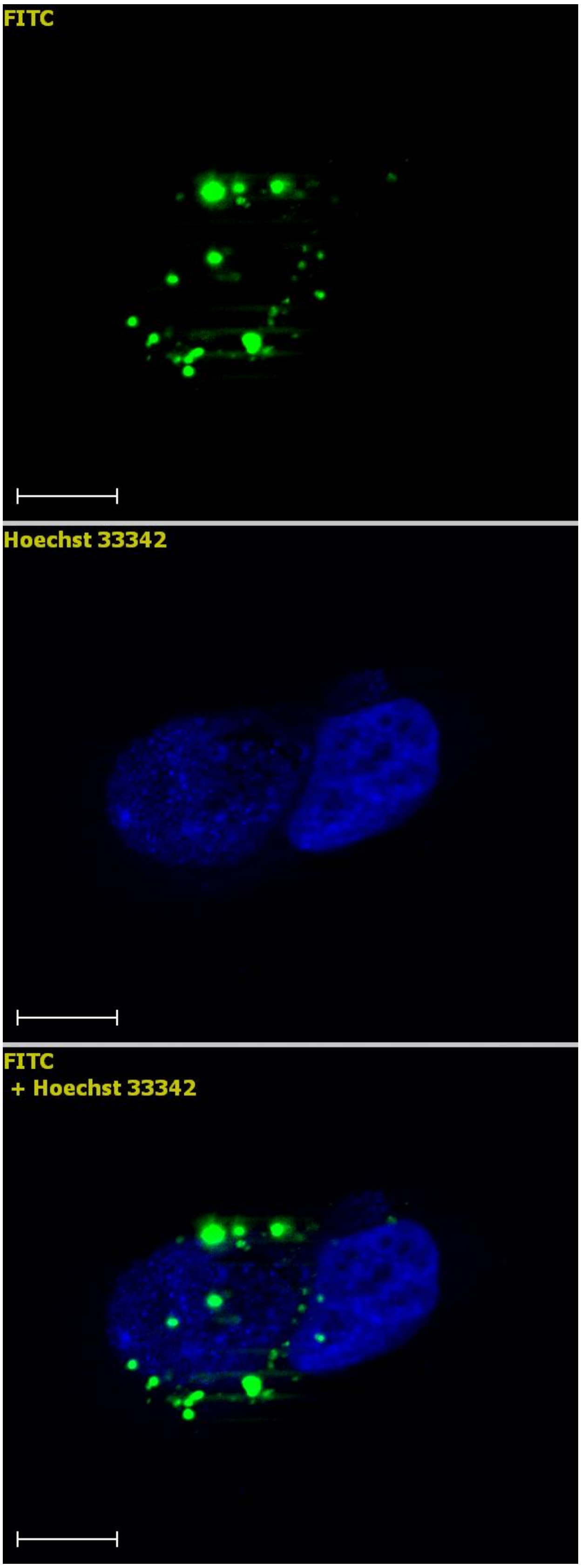

Figure S1. A2EN cell infected with CPP-FITC labeled *Chlamydia trachomatis* elementary bodies (EB)

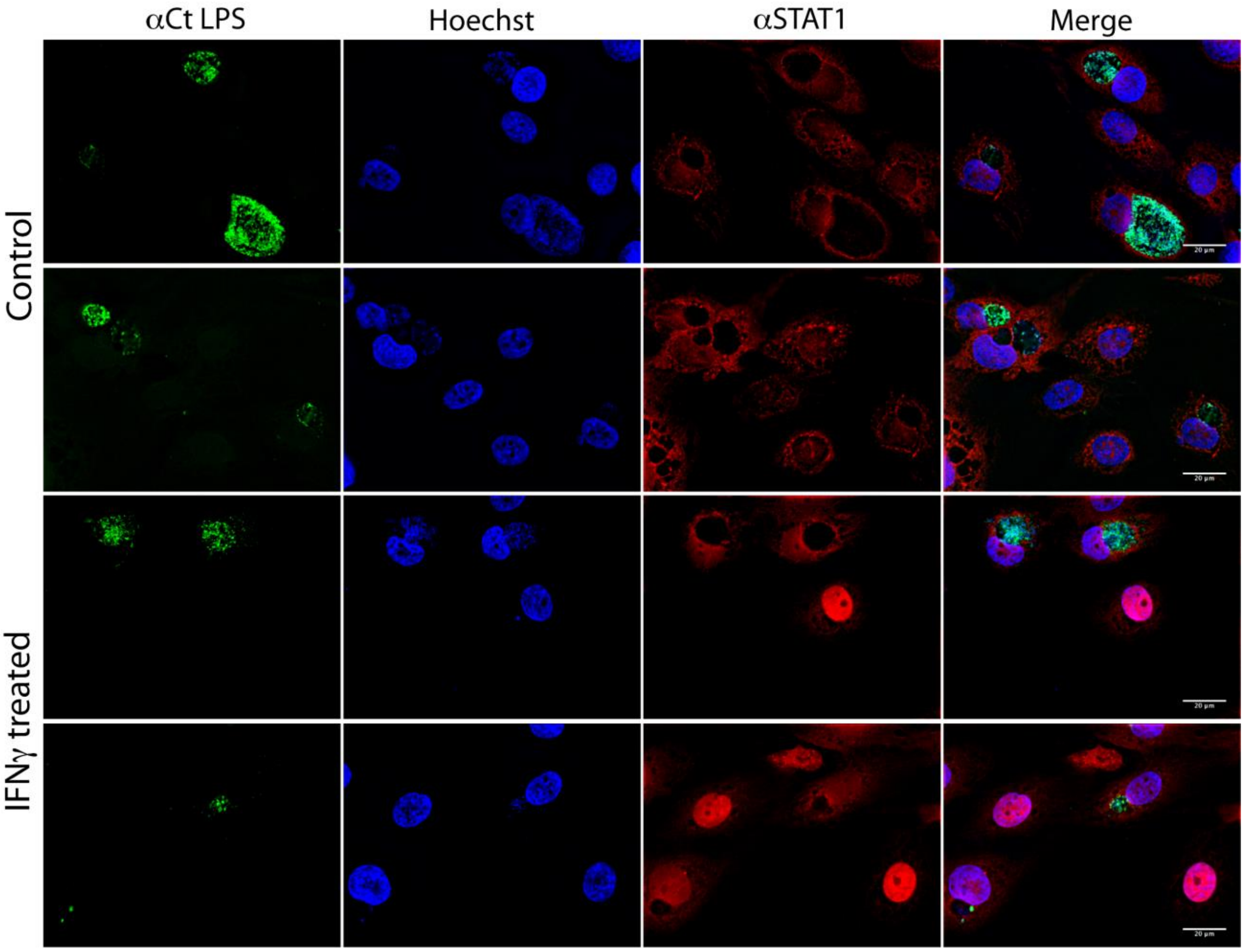

Figure S2. STAT1 localization of *Chlamydia trachomatis*-infected A2EN cells in untreated and IFNγ-treated cell culture monolayers. Chlamydial-LPS (green), nuclei (blue), STAT1 (red).

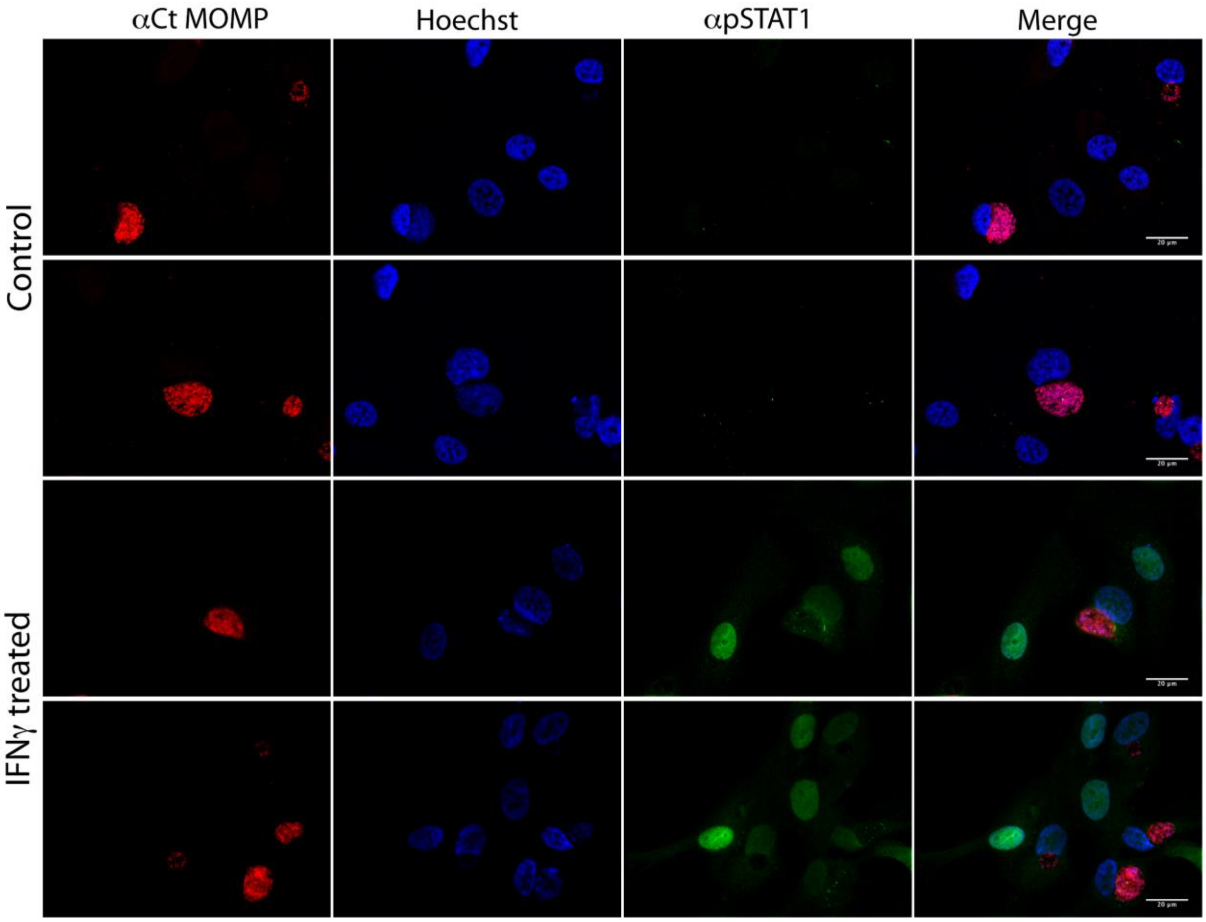

Figure S3. Phosphorylated STAT1 (pSTAT) localization of *Chlamydia trachomatis*-infected A2EN cells in untreated and IFNγ-treated cell culture monolayers using indirect IF staining. Chlmydial MOMP (red), nuclei (blue), pSTAT (green).

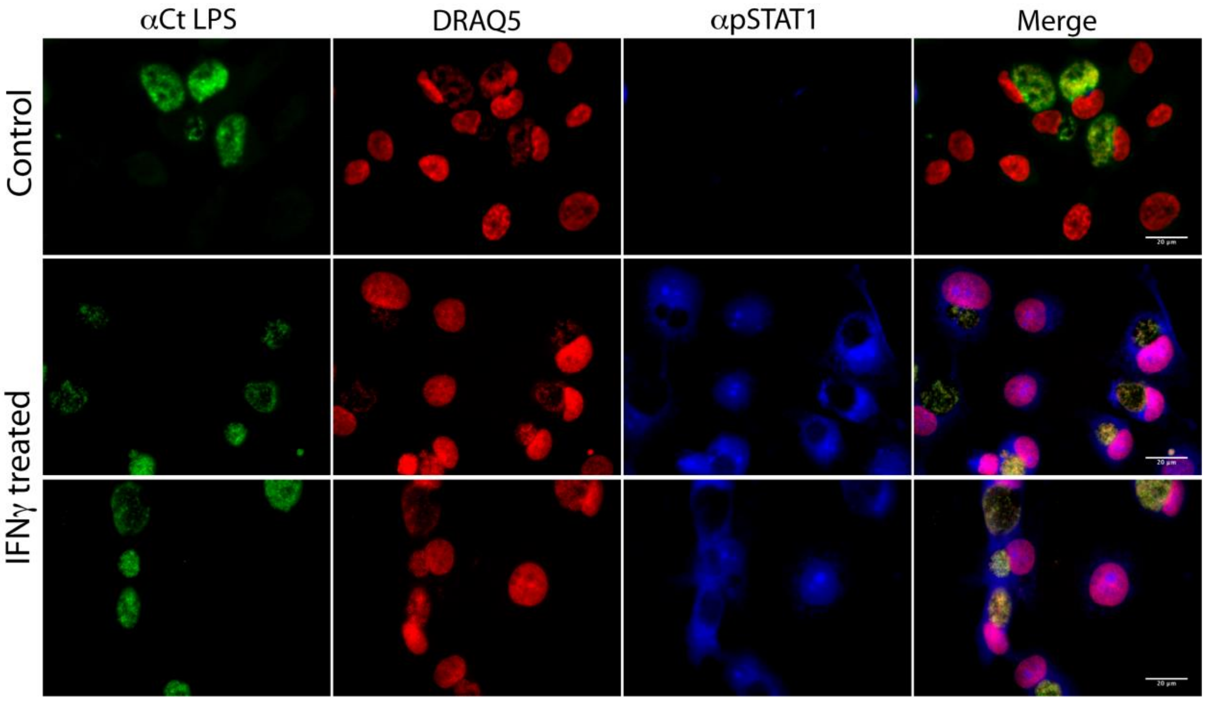

Figure S4. Phosphorylated STAT1 (pSTAT) localization of *Chlamydia trachomatis*-infected A2EN cells in untreated and IFNγ-treated cell culture monolayers using direct IF staining. Chlamydial-LPS (green), nuclei (red), pSTAT (blue).
